# 4-Methyl-2-oxo-2*H*-chromen-7-yl 4-methyl­benzene­sulfonate

**DOI:** 10.1107/S1600536812012238

**Published:** 2012-03-28

**Authors:** Jian-Xin Yang, Hong-Yan Liu, Xiang-Hui Wang

**Affiliations:** a Institute of Materials and Chemical Engineering, Hainan University, Haikou 570228, People’s Republic of China; b School of Chemistry and Chemical Engineering, South China University of Technology, GuangZhou 510640, People’s Republic of China; c Institute of Environmental Science and Engineering, Kunming University of Science and Technology, Kunming 650093, People’s Republic of China

## Abstract

In the title compound, C_17_H_14_O_5_S, the coumarin ring system is nearly planar, with a maximum deviation of 0.034 (2) Å from the mean plane. The dihedral angle between the benzene ring and the coumarin ring system is 56.11 (6)°. The crystal packing is stabilized by C—H⋯O hydrogen bonding, which forms a three-dimensional framework.

## Related literature

For the biological activity of coumarin derivatives, see: Xie *et al.* (2001 ▶); Tanitame *et al.* (2004 ▶); Shao *et al.* (1997 ▶); Rendenbach-Müller *et al.* (1994 ▶); Pochet *et al.* (1996 ▶). For a related structure, see: Yang *et al.* (2007 ▶).
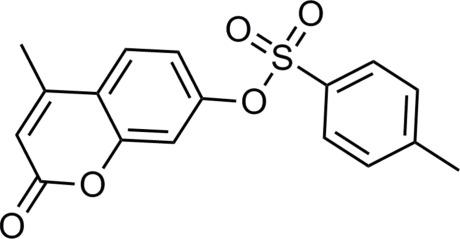


## Experimental

### 

#### Crystal data

C_17_H_14_O_5_S
*M* *_r_* = 330.34
Triclinic, 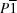

*a* = 7.5582 (19) Å
*b* = 8.024 (2) Å
*c* = 13.336 (4) Å
α = 88.648 (8)°
β = 87.420 (7)°
γ = 74.341 (4)°
*V* = 777.9 (3) Å^3^
*Z* = 2
Mo *K*α radiation
μ = 0.23 mm^−1^
*T* = 153 K
0.54 × 0.41 × 0.40 mm

#### Data collection

Rigaku AFC10/Saturn724+ diffractometer
Absorption correction: multi-scan (*ABSCOR*; Higashi, 1995 ▶) *T*_min_ = 0.885, *T*_max_ = 0.913
8968 measured reflections
4451 independent reflections
3289 reflections with *I* > 2σ(*I*)
*R*_int_ = 0.037

#### Refinement

*R*[*F* ^2^ > 2σ(*F* ^2^)] = 0.042
*wR*(*F* ^2^) = 0.099
*S* = 1.00
4451 reflections
211 parameters
H-atom parameters constrained
Δρ_max_ = 0.30 e Å^−3^
Δρ_min_ = −0.47 e Å^−3^

### 

Data collection: *CrystalClear* (Rigaku, 2008 ▶); cell refinement: *CrystalClear*; data reduction: *CrystalClear*; program(s) used to solve structure: *SHELXS97* (Sheldrick, 2008 ▶); program(s) used to refine structure: *SHELXL97* (Sheldrick, 2008 ▶); molecular graphics: *SHELXTL* (Sheldrick, 2008 ▶) and *DIAMOND* (Brandenburg, 1999 ▶); software used to prepare material for publication: *SHELXTL* and *publCIF* (Westrip, 2010 ▶).

## Supplementary Material

Crystal structure: contains datablock(s) I, global. DOI: 10.1107/S1600536812012238/fy2046sup1.cif

Structure factors: contains datablock(s) I. DOI: 10.1107/S1600536812012238/fy2046Isup2.hkl

Supplementary material file. DOI: 10.1107/S1600536812012238/fy2046Isup3.cml

Additional supplementary materials:  crystallographic information; 3D view; checkCIF report

## Figures and Tables

**Table 1:** Hydrogen-bond geometry (Å, °)

*D*—H⋯*A*	*D*—H	H⋯*A*	*D*⋯*A*	*D*—H⋯*A*
C5—H5⋯O4^i^	0.95	2.50	3.447 (2)	176
C6—H6⋯O3^ii^	0.95	2.49	3.380 (2)	156
C11—H11⋯O3^iii^	0.95	2.50	3.355 (2)	150
C12—H12⋯O2^iv^	0.95	2.58	3.506 (2)	165
C15—H15⋯O5^v^	0.95	2.41	3.284 (2)	152

Symmetry codes: (i) 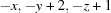; (ii) 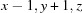; (iii) 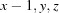; (iv) 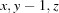; (v) 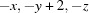.

**Fig. 1.**
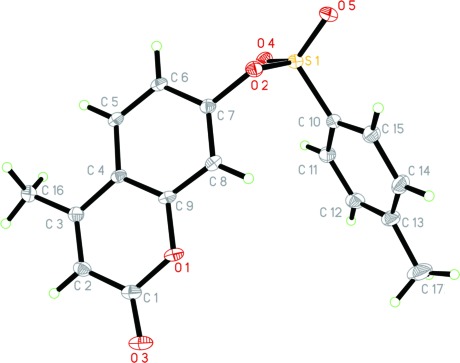
The molecular structure of the title compound with the atom numbering scheme. Displacement ellipsoids are drawn at the 30% probability level. H atoms are presented as a small spheres of arbitrary radius.

**Fig. 2.**
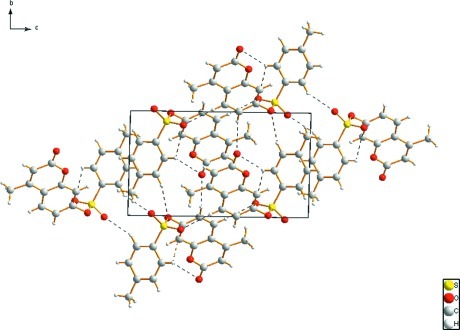
A view of the C—H···O interactions (dotted lines) in the crystal structure of the title compound.

## supplementary crystallographic information

### Comment

Coumarin derivatives exhibit a wide variety of pharmacological activities including anti-HIV (Xie *et al.*, 2001), antibacterial (Tanitame *et al.*, 2004), antioxidant (Shao *et al.*, 1997), antithrombotic (Rendenbach-Müller *et al.*, 1994) and antiinflammatory (Pochet *et al.*, 1996) activities.

The molecular structure is shown in Fig. 1. The dihedral angle between the coumarin ring system and the phenyl ring is 56.11 (6)°. The terminal S═O bond distances of 1.4215 (11) and 1.4219 (11) Å agree with 1.4207 (19) and 1.4331 (19) Å found in a related compound, 4-methyl-7-phenylsulfonamido-2*H*-1-benzopyran-2-one (Yang *et al.*, 2007).

In the crystal the molecules are linked by weak C—H···O hydrogen bonding (Table 1 and Fig. 2).

### Experimental

To a mixture of *para*-toluenesulfonic acid (0.5 g) and acetylacetic ester (10.50 mmol), 4-hydroxyphenyl-4-methylbenzenesulfonate (10.50 mmol) was slowly added at 278–288 K with stirring for 30 min. The reaction mixture was stirred continuously for 12 h at room temperature and then poured into ice–water (100 ml). The solid obtained was filtered off, washed with cold water and dried at room temperature. Colorless crystals of the title compound suitable for X-ray structure analysis were obtained by evaporation of an ethanol solution over a period of two days.

### Refinement

H atoms were placed in calculated positions with C—H = 0.93 Å (aromatic) and 0.96 Å (methyl), and refined in riding mode with *U*_iso_(H) = 1.2 *U*_eq_(C) (aromatic) and *U*_iso_(H) = 1.5 *U*_eq_(C) (methyl).

### Crystal data

**Table:**

C_17_H_14_O_5_S	*Z* = 2
*M**_r_* = 330.34	*F*(000) = 344
Triclinic, *P*1	*D*_x_ = 1.410 Mg m^−^^3^
*a* = 7.5582 (19) Å	Mo *K*α radiation, λ = 0.71073 Å
*b* = 8.024 (2) Å	Cell parameters from 2667 reflections
*c* = 13.336 (4) Å	θ = 2.6–30.0°
α = 88.648 (8)°	µ = 0.23 mm^−^^1^
β = 87.420 (7)°	*T* = 153 K
γ = 74.341 (4)°	Chip, colorless
*V* = 777.9 (3) Å^3^	0.54 × 0.41 × 0.40 mm

### Data collection

**Table:**

Rigaku AFC10/Saturn724+ diffractometer	4451 independent reflections
Radiation source: Rotating Anode	3289 reflections with *I* > 2σ(*I*)
Graphite monochromator	*R*_int_ = 0.037
Detector resolution: 28.5714 pixels mm^-1^	θ_max_ = 30.0°, θ_min_ = 2.8°
φ and ω scans	*h* = −10→10
Absorption correction: multi-scan (*ABSCOR*; Higashi, 1995)	*k* = −11→10
*T*_min_ = 0.885, *T*_max_ = 0.913	*l* = −18→17
8968 measured reflections	

### Refinement

**Table:**

Refinement on *F*^2^	Secondary atom site location: difference Fourier map
Least-squares matrix: full	Hydrogen site location: inferred from neighbouring sites
*R*[*F*^2^ > 2σ(*F*^2^)] = 0.042	H-atom parameters constrained
*wR*(*F*^2^) = 0.099	*w* = 1/[σ^2^(*F*_o_^2^) + (0.0315*P*)^2^ + 0.119*P*] where *P* = (*F*_o_^2^ + 2*F*_c_^2^)/3
*S* = 1.00	(Δ/σ)_max_ < 0.001
4451 reflections	Δρ_max_ = 0.30 e Å^−^^3^
211 parameters	Δρ_min_ = −0.47 e Å^−^^3^
0 restraints	Extinction correction: *SHELXL97* (Sheldrick, 2008), Fc^*^=kFc[1+0.001xFc^2^λ^3^/sin(2θ)]^-1/4^
Primary atom site location: structure-invariant direct methods	Extinction coefficient: 0.0116 (17)

### Special details

**Table:**

Geometry. All e.s.d.'s (except the e.s.d. in the dihedral angle between two l.s. planes) are estimated using the full covariance matrix. The cell e.s.d.'s are taken into account individually in the estimation of e.s.d.'s in distances, angles and torsion angles; correlations between e.s.d.'s in cell parameters are only used when they are defined by crystal symmetry. An approximate (isotropic) treatment of cell e.s.d.'s is used for estimating e.s.d.'s involving l.s. planes.
Refinement. Refinement of *F*^2^ against ALL reflections. The weighted *R*-factor *wR* and goodness of fit *S* are based on *F*^2^, conventional *R*-factors *R* are based on *F*, with *F* set to zero for negative *F*^2^. The threshold expression of *F*^2^ > σ(*F*^2^) is used only for calculating *R*-factors(gt) *etc*. and is not relevant to the choice of reflections for refinement. *R*-factors based on *F*^2^ are statistically about twice as large as those based on *F*, and *R*- factors based on ALL data will be even larger.

### Fractional atomic coordinates and isotropic or equivalent isotropic displacement parameters (Å^2^)

**Table:**

	*x*	*y*	*z*	*U*_iso_*/*U*_eq_	
S1	−0.05701 (5)	0.90186 (5)	0.20148 (3)	0.02605 (11)	
O1	0.63814 (14)	0.58774 (12)	0.35269 (7)	0.0263 (2)	
O2	0.11089 (15)	0.98205 (12)	0.22320 (7)	0.0270 (2)	
O3	0.88672 (15)	0.39807 (14)	0.40502 (9)	0.0362 (3)	
O4	−0.14270 (15)	0.87024 (14)	0.29442 (7)	0.0324 (3)	
O5	−0.15830 (17)	1.02006 (14)	0.13018 (8)	0.0384 (3)	
C1	0.7537 (2)	0.51511 (19)	0.42877 (12)	0.0276 (3)	
C2	0.7040 (2)	0.58301 (19)	0.52862 (12)	0.0276 (3)	
H2	0.7814	0.5339	0.5819	0.033*	
C3	0.5524 (2)	0.71307 (18)	0.54922 (11)	0.0240 (3)	
C4	0.43676 (19)	0.79009 (17)	0.46707 (10)	0.0211 (3)	
C5	0.2782 (2)	0.92868 (17)	0.47851 (11)	0.0227 (3)	
H5	0.2448	0.9795	0.5426	0.027*	
C6	0.1701 (2)	0.99233 (17)	0.39833 (10)	0.0237 (3)	
H6	0.0638	1.0872	0.4064	0.028*	
C7	0.2198 (2)	0.91503 (17)	0.30561 (10)	0.0222 (3)	
C8	0.3765 (2)	0.78118 (18)	0.28978 (10)	0.0242 (3)	
H8	0.4094	0.7319	0.2253	0.029*	
C9	0.48388 (19)	0.72125 (17)	0.37112 (11)	0.0218 (3)	
C10	0.0542 (2)	0.70422 (18)	0.14554 (10)	0.0237 (3)	
C11	0.0484 (2)	0.55129 (19)	0.19411 (12)	0.0301 (3)	
H11	−0.0192	0.5528	0.2560	0.036*	
C12	0.1434 (2)	0.3959 (2)	0.15060 (13)	0.0363 (4)	
H12	0.1384	0.2902	0.1828	0.044*	
C13	0.2454 (2)	0.3912 (2)	0.06117 (13)	0.0367 (4)	
C14	0.2448 (2)	0.5469 (2)	0.01312 (12)	0.0373 (4)	
H14	0.3111	0.5456	−0.0492	0.045*	
C15	0.1497 (2)	0.7033 (2)	0.05420 (11)	0.0310 (3)	
H15	0.1497	0.8089	0.0204	0.037*	
C16	0.4986 (2)	0.7756 (2)	0.65416 (11)	0.0307 (3)	
H16A	0.5894	0.7093	0.7004	0.037*	
H16B	0.3773	0.7599	0.6731	0.037*	
H16C	0.4938	0.8987	0.6575	0.037*	
C17	0.3556 (3)	0.2207 (2)	0.01848 (17)	0.0579 (6)	
H17A	0.4712	0.1806	0.0533	0.069*	
H17B	0.3824	0.2354	−0.0533	0.069*	
H17C	0.2849	0.1351	0.0276	0.069*	

### Atomic displacement parameters (Å^2^)

**Table:**

	*U*^11^	*U*^22^	*U*^33^	*U*^12^	*U*^13^	*U*^23^
S1	0.0268 (2)	0.02155 (18)	0.0275 (2)	−0.00202 (14)	−0.00334 (15)	−0.00134 (14)
O1	0.0219 (5)	0.0191 (5)	0.0340 (6)	0.0000 (4)	0.0056 (4)	−0.0021 (4)
O2	0.0322 (6)	0.0196 (5)	0.0294 (5)	−0.0069 (4)	−0.0047 (4)	0.0032 (4)
O3	0.0241 (6)	0.0242 (5)	0.0549 (7)	0.0013 (5)	0.0050 (5)	0.0014 (5)
O4	0.0301 (6)	0.0361 (6)	0.0309 (6)	−0.0090 (5)	0.0059 (5)	−0.0072 (5)
O5	0.0431 (7)	0.0268 (6)	0.0391 (6)	0.0033 (5)	−0.0153 (5)	0.0017 (5)
C1	0.0197 (7)	0.0198 (7)	0.0428 (9)	−0.0054 (6)	0.0024 (6)	0.0019 (6)
C2	0.0213 (7)	0.0235 (7)	0.0384 (8)	−0.0064 (6)	−0.0035 (6)	0.0024 (6)
C3	0.0211 (7)	0.0207 (7)	0.0320 (8)	−0.0086 (6)	−0.0010 (6)	−0.0003 (6)
C4	0.0191 (7)	0.0160 (6)	0.0287 (7)	−0.0058 (5)	0.0022 (6)	−0.0026 (5)
C5	0.0215 (7)	0.0186 (6)	0.0278 (7)	−0.0053 (5)	0.0036 (6)	−0.0053 (5)
C6	0.0212 (7)	0.0170 (6)	0.0320 (8)	−0.0036 (5)	0.0025 (6)	−0.0042 (6)
C7	0.0248 (7)	0.0168 (6)	0.0259 (7)	−0.0070 (6)	−0.0007 (6)	0.0010 (5)
C8	0.0272 (8)	0.0197 (7)	0.0252 (7)	−0.0062 (6)	0.0044 (6)	−0.0035 (5)
C9	0.0181 (7)	0.0150 (6)	0.0312 (7)	−0.0035 (5)	0.0055 (6)	−0.0022 (5)
C10	0.0254 (7)	0.0210 (7)	0.0240 (7)	−0.0046 (6)	−0.0025 (6)	−0.0014 (5)
C11	0.0331 (9)	0.0256 (8)	0.0317 (8)	−0.0080 (7)	−0.0019 (7)	0.0017 (6)
C12	0.0378 (10)	0.0211 (7)	0.0501 (10)	−0.0073 (7)	−0.0097 (8)	0.0008 (7)
C13	0.0276 (8)	0.0295 (8)	0.0517 (10)	−0.0031 (7)	−0.0080 (8)	−0.0139 (7)
C14	0.0322 (9)	0.0449 (10)	0.0327 (9)	−0.0066 (8)	0.0051 (7)	−0.0129 (7)
C15	0.0352 (9)	0.0282 (8)	0.0282 (8)	−0.0066 (7)	0.0009 (7)	0.0013 (6)
C16	0.0311 (9)	0.0295 (8)	0.0319 (8)	−0.0084 (7)	−0.0042 (7)	−0.0027 (6)
C17	0.0407 (11)	0.0384 (10)	0.0896 (16)	0.0010 (9)	−0.0060 (11)	−0.0315 (10)

### Geometric parameters (Å, º)

**Table:**

S1—O5	1.4215 (11)	C8—C9	1.3824 (19)
S1—O4	1.4219 (11)	C8—H8	0.9500
S1—O2	1.6097 (11)	C10—C11	1.384 (2)
S1—C10	1.7485 (15)	C10—C15	1.387 (2)
O1—C9	1.3710 (16)	C11—C12	1.386 (2)
O1—C1	1.3817 (18)	C11—H11	0.9500
O2—C7	1.4104 (16)	C12—C13	1.386 (2)
O3—C1	1.2119 (18)	C12—H12	0.9500
C1—C2	1.446 (2)	C13—C14	1.389 (2)
C2—C3	1.348 (2)	C13—C17	1.507 (2)
C2—H2	0.9500	C14—C15	1.379 (2)
C3—C4	1.4521 (19)	C14—H14	0.9500
C3—C16	1.499 (2)	C15—H15	0.9500
C4—C9	1.3983 (19)	C16—H16A	0.9800
C4—C5	1.4028 (19)	C16—H16B	0.9800
C5—C6	1.3782 (19)	C16—H16C	0.9800
C5—H5	0.9500	C17—H17A	0.9800
C6—C7	1.3868 (19)	C17—H17B	0.9800
C6—H6	0.9500	C17—H17C	0.9800
C7—C8	1.379 (2)		
			
O5—S1—O4	120.25 (8)	O1—C9—C4	121.76 (13)
O5—S1—O2	103.06 (6)	C8—C9—C4	122.18 (13)
O4—S1—O2	109.02 (6)	C11—C10—C15	121.11 (14)
O5—S1—C10	110.90 (7)	C11—C10—S1	119.45 (12)
O4—S1—C10	109.26 (7)	C15—C10—S1	119.43 (11)
O2—S1—C10	102.75 (6)	C10—C11—C12	118.67 (15)
C9—O1—C1	121.11 (12)	C10—C11—H11	120.7
C7—O2—S1	117.90 (8)	C12—C11—H11	120.7
O3—C1—O1	116.22 (14)	C11—C12—C13	121.42 (15)
O3—C1—C2	126.26 (15)	C11—C12—H12	119.3
O1—C1—C2	117.51 (13)	C13—C12—H12	119.3
C3—C2—C1	122.73 (14)	C12—C13—C14	118.46 (15)
C3—C2—H2	118.6	C12—C13—C17	120.29 (17)
C1—C2—H2	118.6	C14—C13—C17	121.25 (17)
C2—C3—C4	118.39 (13)	C15—C14—C13	121.24 (16)
C2—C3—C16	121.80 (14)	C15—C14—H14	119.4
C4—C3—C16	119.79 (13)	C13—C14—H14	119.4
C9—C4—C5	117.80 (13)	C14—C15—C10	119.03 (15)
C9—C4—C3	118.45 (13)	C14—C15—H15	120.5
C5—C4—C3	123.74 (13)	C10—C15—H15	120.5
C6—C5—C4	121.12 (13)	C3—C16—H16A	109.5
C6—C5—H5	119.4	C3—C16—H16B	109.5
C4—C5—H5	119.4	H16A—C16—H16B	109.5
C5—C6—C7	118.61 (13)	C3—C16—H16C	109.5
C5—C6—H6	120.7	H16A—C16—H16C	109.5
C7—C6—H6	120.7	H16B—C16—H16C	109.5
C8—C7—C6	122.58 (13)	C13—C17—H17A	109.5
C8—C7—O2	118.74 (12)	C13—C17—H17B	109.5
C6—C7—O2	118.60 (13)	H17A—C17—H17B	109.5
C7—C8—C9	117.65 (13)	C13—C17—H17C	109.5
C7—C8—H8	121.2	H17A—C17—H17C	109.5
C9—C8—H8	121.2	H17B—C17—H17C	109.5
O1—C9—C8	116.05 (12)		
			
O5—S1—O2—C7	167.92 (10)	C1—O1—C9—C4	0.17 (19)
O4—S1—O2—C7	39.08 (11)	C7—C8—C9—O1	179.78 (12)
C10—S1—O2—C7	−76.75 (11)	C7—C8—C9—C4	1.0 (2)
C9—O1—C1—O3	−179.83 (12)	C5—C4—C9—O1	178.86 (12)
C9—O1—C1—C2	1.12 (19)	C3—C4—C9—O1	−2.0 (2)
O3—C1—C2—C3	−179.45 (15)	C5—C4—C9—C8	−2.4 (2)
O1—C1—C2—C3	−0.5 (2)	C3—C4—C9—C8	176.75 (12)
C1—C2—C3—C4	−1.3 (2)	O5—S1—C10—C11	−135.28 (13)
C1—C2—C3—C16	176.97 (13)	O4—S1—C10—C11	−0.47 (14)
C2—C3—C4—C9	2.54 (19)	O2—S1—C10—C11	115.18 (12)
C16—C3—C4—C9	−175.78 (13)	O5—S1—C10—C15	45.99 (14)
C2—C3—C4—C5	−178.39 (13)	O4—S1—C10—C15	−179.20 (12)
C16—C3—C4—C5	3.3 (2)	O2—S1—C10—C15	−63.55 (13)
C9—C4—C5—C6	1.5 (2)	C15—C10—C11—C12	1.2 (2)
C3—C4—C5—C6	−177.61 (13)	S1—C10—C11—C12	−177.49 (12)
C4—C5—C6—C7	0.8 (2)	C10—C11—C12—C13	1.1 (2)
C5—C6—C7—C8	−2.3 (2)	C11—C12—C13—C14	−2.6 (2)
C5—C6—C7—O2	−178.95 (12)	C11—C12—C13—C17	176.63 (15)
S1—O2—C7—C8	92.71 (14)	C12—C13—C14—C15	1.8 (2)
S1—O2—C7—C6	−90.55 (13)	C17—C13—C14—C15	−177.42 (16)
C6—C7—C8—C9	1.5 (2)	C13—C14—C15—C10	0.4 (2)
O2—C7—C8—C9	178.07 (12)	C11—C10—C15—C14	−2.0 (2)
C1—O1—C9—C8	−178.66 (12)	S1—C10—C15—C14	176.72 (12)

### Hydrogen-bond geometry (Å, º)

**Table:**

*D*—H···*A*	*D*—H	H···*A*	*D*···*A*	*D*—H···*A*
C5—H5···O4^i^	0.95	2.50	3.447 (2)	176
C6—H6···O3^ii^	0.95	2.49	3.380 (2)	156
C11—H11···O3^iii^	0.95	2.50	3.355 (2)	150
C12—H12···O2^iv^	0.95	2.58	3.506 (2)	165
C15—H15···O5^v^	0.95	2.41	3.284 (2)	152

Symmetry codes: (i) −*x*, −*y*+2, −*z*+1; (ii) *x*−1, *y*+1, *z*; (iii) *x*−1, *y*, *z*; (iv) *x*, *y*−1, *z*; (v) −*x*, −*y*+2, −*z*.
